# Structure and Photoluminescence Properties of Rare-Earth (Dy^3+^, Tb^3+^, Sm^3+^)-Doped BaWO_4_ Phosphors Synthesized via Co-Precipitation for Anti-Counterfeiting

**DOI:** 10.3390/ma13184165

**Published:** 2020-09-19

**Authors:** Jae-yong Jung, Juna Kim, Young-Seok Shim, Donghyun Hwang, Chang Sik Son

**Affiliations:** 1 Division of Materials Science and Engineering, Silla University, Busan 46958, Korea; eayoung21@naver.com (J.-y.J.); ysshim@silla.ac.kr (Y.-S.S.); 2 School of Materials Science and Engineering, Pusan National University, Busan 46241, Korea; kja6037@pusan.ac.kr

**Keywords:** BaWO_4_, anti-counterfeiting, photoluminescence, co-precipitation

## Abstract

Barium tungstate (BaWO_4_) powders with various sintering temperatures, and BaWO_4_:Dy^3+^ phosphor samples with concentrations of different rare-earth (RE) activator ions (Dy^3+^, Sm^3+^, Tb^3+^) were prepared through co-precipitation. The structural, morphological, and photoluminescent characteristics of barium tungstate phosphors depend on the concentration of RE ions. The crystallographic characteristics of the synthesized BaWO_4_ were analyzed using X-ray diffraction (XRD) patterns. The size and shape of the crystalline particles were estimated based on images measured with a field emission scanning electron microscope (FE-SEM). As the sintering temperature of the BaWO_4_ particles increased from 400 °C to 1000 °C, the size of the particles gradually increased and showed a tendency to clump together. In the sample doped with 7 mol % Dy^3+^ ions, the intensity of all emission bands reached their maximum. The emission spectra of the RE^3+^-doped BaWO_4_ powders by excitation at 325 nm were composed of yellow (Dy^3+^), red (Sm^3^+), and green (Tb^3+^) band at 572, 640, and 544 nm. This indicates that most of the RE^3+^ ions absorbed the position without reversal symmetry in the BaWO_4_ lattice. These results propose that strong emission intensity and tunable color for the phosphors can be accomplished by rare-earth doped host with an suitable quantity. In addition, the phosphor thin films, having high transparency from aqueous colloidal solutions, were deposited on banknotes, and it is considered whether it is suitable for anti-counterfeiting applications.

## 1. Introduction

Rare-earth (RE) ion activated metal tungstates (MXO_4_, M = Ca, Ba or Sr, X = W or Mo) have excellent luminescence properties as well as high chemical and thermal stability. Therefore, various studies that can be used in broad fields such as solar cells, solid-state lasers, white-light emitting diodes (w-LEDs), and display are being conducted [1,2,3,4,5]. Among these tungstates, tetragonal scheelite-like structure barium tungstate (BaWO_4_) is an encouraging luminescent host material for phosphors, because the ${\mathrm{WO}}_{4}^{2-}$ group indicates high absorption in the ultraviolet (UV) and the blue ranges; this generates particular emission bands by energy transfer from the ${\mathrm{WO}}_{4}^{2-}$ groups to the RE ions [6,7,8]. These properties of BaWO_4_ are sufficiently applicable to the anticounterfeiting technology.

Representative anti-counterfeiting materials that have recently been in the spotlight include phosphors, organic dyes, and quantum dots. Such materials can be appropriately used in the aspect of protecting documents or industrial products with functions that are difficult to duplicate [9,10]. In particular, the application of fluorescent materials with high transparency can conceal information encryption and realize dynamic anti-counterfeiting. Inorganic phosphors have distinguished properties such as high luminescence intensity, sustainability, and inertness under sunlight. BaWO_4_ has been regarded as a promising candidate for fluorescence anti-counterfeiting materials among a variety of inorganic phosphors that meet these points [11,12,13,14,15]. Many studies have been performed to develop synthesized BaWO_4,_ using various techniques such as solid-state reaction [16,17], Czochralski crystal growth [18,19], cell electrochemical technique [20], and polymeric precursor method [21]. X. Sun et al. reported on the luminescence properties of BaWO_4_:Ln^3+^ (Ln = Eu, Tb, and Dy) powders synthesized through the traditional solid-state reaction method, which showed red, green, and yellow emissions [22]. Thongtem et al. reported the use of microwave radiation in a solvothermal process as a method to accelerate the formation of tungstates and molybdates, which have a scheelite-type structure [23,24]. Zhang et al. reported fishbone-like nanoassembled BaWO_4_ structures, which could be prepared using a reverse-phase microemulsion method [25]. 

In this work, the synthesis of BaWO_4_ crystal powders with various sintering temperatures and the effect of RE ion concentrations on the structure and photoluminescent characteristics of BaWO_4_:Dy^3+^ phosphor powders through co-precipitation are described. Properties such as the optimal sintering temperature and doping concentration for the yellow emission light were examined. Finally, RE^3+^ (RE = Dy, Tb, Sm)-doped BaWO_4_ was synthesized for anti-counterfeiting application. We showed three colors of RE^3+^-doped BaWO_4_ phosphors—yellow, green, and red—deposited on bank notes to demonstrate the phosphors’ thin films, which can realistically be applied in anti-counterfeiting due to their concealment ability.

## 2. Materials and Methods 

### 2.1. Synthesis of BaWO_4_ Powders and BaWO_4_:RE^3+^ Phosphors

BaWO_4_ powders and BaWO_4_:RE^3+^ (RE = Dy, Tb, and Sm) phosphors were prepared via co-precipitation with Dy^3+^ ( *x* = 1–40 mol %), 7 mol % Tb^3+^, and 7 mol % Sm^3+^ concentrations. Barium acetate ((CH_3_COO)_2_Ba), sodium tungstate (Na_2_WO_4_), dysprosium(Ⅲ) nitrate hydrate (Dy(NO_3_)_3_·xH_2_O, Dy^3+^), samarium(Ⅲ) nitrate hydrate(Sm(NO_3_)_3_·xH_2_O, Sm^3+^), and terbium(Ⅲ) nitrate hydrate (Tb(NO_3_)_3_·xH_2_O, Tb^3+^) were applied by appropriate stoichiometric ratios of starting regents. Firstly, barium acetate and sodium tungstate were dissolved in two separate beakers containing 50 mL of deionized water to form solution A, which was constantly stirred until it reached 80 °C. Next, the sodium tungstate dihydrate solution B was added to the solution A, and maintained under agitation at 80 °C for 20 min. Finally, the white powdery precipitate was rapidly formed by this process, and the resulting precipitate was rinsed several times with deionized water.

The white powders were dried in an oven at 80 °C overnight [26]. The precursors were putted in alumina crucibles. The precursors were then calcined in a furnace at 400 °C for 3 h. After calcination, the samples were sintered for 5 h at different temperatures of 400, 600, 800, 900, and 1000 °C, respectively. Thereafter, the sintering temperature was cooled down to room temperature. In addition, in order to realize the photoluminescence properties of Dy^3+^-doped BaWO_4_, precursors were prepared using the same procedure, by adding dysprosium nitrate to the solution and dissolving according to the various molar ratios (1, 2.5, 5, 7, 10, 20, 40 mol %). Each precursor was calcined at 400 °C for 3 h, sintered at 900 °C for 5 h, and then cooled down to room temperature. Finally, phosphors doped with terbium nitrate (7 mol %) and samarium nitrate (7 mol %) were prepared using the same procedure.

### 2.2. Chraraterization

The crystallographic characteristics of BaWO_4_ powders and BaWO_4_:RE^3+^ (RE = Dy, Tb, Sm) phosphors were examined by X-ray diffraction (XRD; Rigaku Ultima IV, Tokyo, Japan). The chemical composition and oxidation state of the synthesized phosphors were investigated by X-ray photoelectron spectroscopy (XPS, ESCALAB 250XI, Waltham, MA, USA). The peak position of the insulating samples was calibrated using the C1 of 285 eV. Raman spectra were analyzed by Raman spectrometer (LabRam-HR 800, Horiba Jobin-Yvon, France), equipped with a 633 nm He–Ne laser as the excitation source. The surface morphology and microstructure were observed by field emission scanning electron microscope (FE-SEM, SU-8220, Hitach, Tokyo, Japan). The photoluminescence spectra were obtained through a photomultiplier tube operating at 350 V and a fluorescence spectrophotometer (Scinco, FS-2, Seoul, Korea).

### 2.3. BaWO_4_:RE^3+^ Phorsphors for Anti-Counterfeiting Application

First of all, the oily dirt from the fingers was washed with soapy water and air-dried to remove it. After that, the fingerprints of the index finger were marked on the surfaces of several glass substrates. As a final step, the RE^3+^ (RE = Dy, Tb, Sm) doped BaWO_4_ phosphors were applied to the glass substrate surfaces and the latent fingerprints of the surfaces were carefully wiped off. The excess phosphor remaining on the surface was removed using a light feather brush. The latent fingerprints coated with the RE^3+^-doped BaWO_4_ phosphors were developed using a UV lamp with a wavelength of 254 nm, and the appearance of the fingerprints was confirmed by photographing. The solution for the anti-counterfeiting function was prepared as a colloidal solution containing 1 wt % of solids by dispersing RE^3+^-doped BaWO_4_ phosphors in an aqueous solution containing 10 wt % of polyvinylpyrrolidone (PVP, M.W. = 14,000). The solution was printed on banknotes with a brush and then dried at 80 °C for 1 h. The colors of the phosphor thin films were confirmed using the UV lamp [27].

## 3. Results

### 3.1. Structure and Photoluminescence Properties of BaWO_4_ and Dy^3+^ Doped BaWO_4_

**Figure 1:**
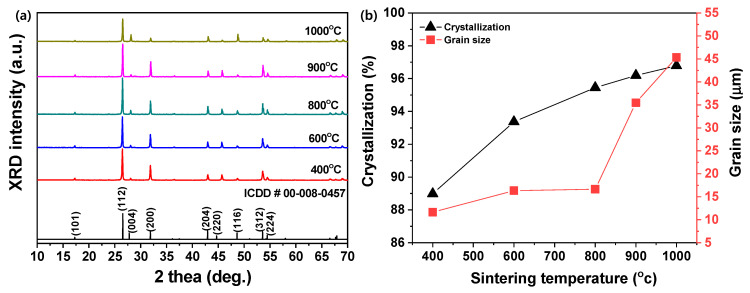
(**a**) XRD patterns and (**b**) crystallization and grain size of BaWO_4_ powders with various sintering temperatures (400, 600, 800, 900, 1000 °C).

Figure 1a shows the XRD patterns and Figure 1b shows crystallization and grain size of BaWO_4_ powders. The peaks of BaWO_4_ were observed at various sintering temperature conditions. 

The main peak (112) phase showed the strongest signal, and the other signals also matched International Center for Diffraction data (ICDD # 00-008-0457). As shown in Figure 1a, two main diffraction peaks appeared in 2θ = 26.48° and 31.88°, which represented the patterns from the (112) and (200) planes of BaWO_4_. The peaks in the XRD patterns represent the crystal structure of the body-center primordial tetragonal scheelite phases [28,29]. The crystallinity and grain size of the BaWO_4_ were calculated using the (112) phase as shown in Figure 1b. On this occasion, crystallinity was calculated using Equation (1) as follows:

(1) $$\mathrm{Crystallinity}\;(\%)\;=\left(I_{T}-I_{A}\right)\times 100$$

where I_T_ is total area of all peaks and I_A_ is area of amorphous peaks from XRD patterns [30]. The area was calculated using Origin Pro 2018 software.

It was shown that the crystallinity increased as sintering temperature increased. However, crystallization did not significantly change at calcination temperatures of 900 to 1000 °C. In addition, the particle size was calculated by substituting full width at half maximum (FWHM) and peak position on the main peak (112) plane in XRD patterns using Scherrer’s equation as follows:

(2) d=kλ/βcosθ

where d = the average crystallite size; k = Scherrer constant (0.9); λ = X-ray wavelength (Cu = 0.15406 nm); β = FWHM, which has to be converted to radians; θ = angle of diffraction [31]. As with the crystallinity, it was shown that particle size increased as the sintering temperature increased.

**Figure 2:**
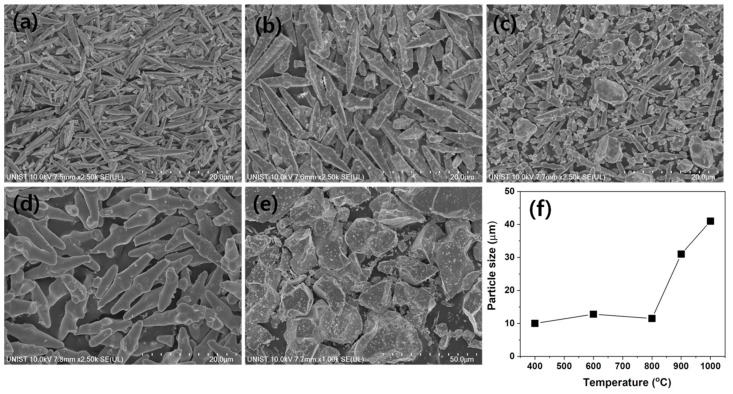
FE-SEM images of BaWO_4_ powders with various sintering temperatures: (**a**) 400, (**b**) 600, (**c**) 800, (**d**) 900, (**e**) 1000 °C, (**f**) particle size.

Shape and grain size of the synthesized BaWO_4_ powders were measured, and the FE-SEM images are shown in Figure 2.

The FE-SEM images of the BaWO_4_ powders show that it was composed of the particles with an octahedral-like shape. The particle sizes of the BaWO_4_ powders increased from about 10 to 43 μm, with increasing sintering temperatures as shown in Figure 2f. 

Generally, in the literature, the formation of BaWO_4_ is usually observed as shapes and templates like octahedrons [32]. M. Oliveria et al. showed the local coordination (clusters) of Ba and W atoms on the surfaces of (001), (101), (110), (100), (111), and (112). One of the most obvious differences in the structural properties between the body and the surface is the reduction in the coordination of oxygen (O) atoms in the top layer. This reduction in the coordination of the O atoms is reported to be due to the change in the O value by vacancies that create gaps between adjacent layers [33]. In addition, Gao et al. reported that the morphology of the scheelite crystals mainly present on the exposed (112), (001), and (100) surfaces, with the (112) surface being the most stable [34,35].

**Figure 3:**
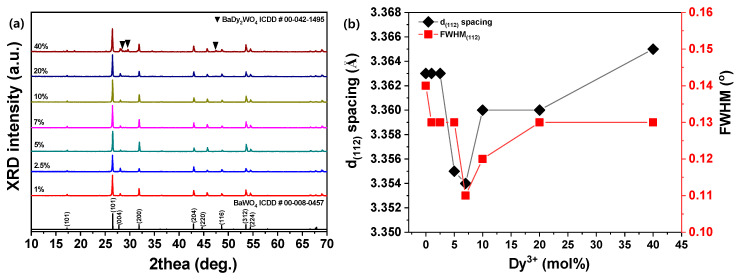
(**a**) XRD patterns and (**b**) lattice constant d_(112)_ spacing and full width at half maximum (FWHM) of Dy^3+^-doped BaWO_4_ samples with various rare-earth ions.

In this work, it was shown that the sintering temperature changed particle shapes sharply to octahedron shapes. In samples sintered at 1000 °C, overgrowth and an uneven particle distribution resulted in excessive grain growth. As a result, a sintering temperature of 900 °C, in which the average particle size was uniform, was selected to synthesize BaWO_4_ phosphor-doped Dy^3+^ ions. The XRD patterns of BaWO_4_ phosphors, according to various doped Dy^3+^, are shown in Figure 3a, and the change of lattice constant through the main peak (112) plane is shown in Figure 3b.

There were no phases detected for the activator ions, indicating that the Dy^3+^ ions had no effect on the BaWO_4_ phase composition but slightly changed the position where the main peak (112) plane was detected compared to the undoped BaWO_4_ powders, In addition, 40 mol % doped BaWO_4_ phosphors showed secondary phase of BaDy_2_O_4_, according to the XRD signals. Lattice constants were determined as d_(112)_ spacing = 3.363 Å for BaWO_4,_ and a small amount of Dy^3+^ (<2.5 mol %) was added. Further doping of Dy^3+^ (≤7 mol %) decreased d_(112)_ spacing (3.363 → 3.354 Å) and FWHM (0.14 → 0.11°). The ionic radii for each cations are different, such as r (Dy^3+^) = 1.03 Å, r (Ba^2+^) = 1.142 Å and r (W^6+^) = 0.74 Å. Therefore, it is possible for Dy^3+^ ions to be substituted with Ba^2+^ ions in the BaWO_4_ structure [36,37]. However, as doping of Dy^3+^ was increased, the lattice constant and FWHM increased. Here, a small amount of Dy^3+^ doping improved crystallization of BaWO_4,_ but a large amount of doping formed the secondary phase.

**Figure 4:**
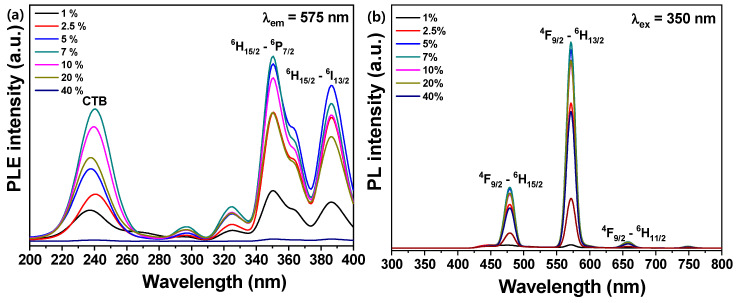
Photoluminescent properties of Dy^3+^-doped BaWO_4_ phosphors with various concentrations of activator ions: (**a**) photoluminescence excitation, and (**b**) photoluminescence.

Figure 4a,b show the photoluminescence excitation (PLE) and photoluminescence (PL) spectra of the BaWO_4_:Dy^3+^ phosphors synthesized with various dysprosium ions doping concentrations.

In the PLE spectrum shown in Figure 4a, the peak intensities for the excitation wavelength of 350 nm represent the ^6^H_15/2_ → ^4^P_7/2_ level of the Dy^3+^ ion. The broad peak patterns shown in the excitation wavelength range from 220 to 260 nm, and correspond to the Dy^3+^-O^2-^ charge transfer band (CTB) of the host crystal [38,39,40]. The PLE intensities of all the excitation bands in Figure 4a were dramatically improved in proportion to the concentration ratio of Dy^3+^ ions, changing from 1 to 5 mol %. The PLE intensities reached their maximum when the concentration of Dy^3+^ ion was 7 mol %, and the intensities decreased significantly as the concentration increased from 10 to 40 mol %. As a result of the PL spectra in Figure 4b, measured at an excitation wavelength of 350 nm, two clearly distinct emission bands were observed at the specific wavelengths of 479 nm and 572 nm. These bands are known to correspond to the ^4^F_9/2_ → ^6^H_15/2_ magnetic dipole transitions and the ^4^F_9/2_ → ^6^H_13/2_ electric dipole transitions, respectively [41]. The intensity ratios of the ^4^F_9/2_ → ^6^H_13/2_ (572 nm) ED to the ^4^F_9/2_ → ^6^H_15/2_ (479 nm) MD transitions were estimated to be approximately 3.29, 3.32, 3.36, 3.46, and 3.39 for the BaWO_4_ powders synthesized with 1, 2.5, 5, 7, and 10 mol % Dy^3+^, respectively. According to the results, the positions of the Dy^3+^ ions in the BaWO_4_ lattice was displaced from the position without inverse symmetry to the sites and with inverse symmetry at the Dy^3+^ concentration of 20 mol %. The Dy^3+^ ions were greatly yellow. Emission appeared from the ^4^F_9/2_ → ^6^H_13/2_ electric dipole transition, caused by 7 mol % Dy^3+^. The change in Dy^3+^ ions mole fraction was increased from 10 to 40 mol %, and the intensity of the dominant ^4^F_9/2_ → ^6^H_13/2_ transition decreased rapidly owing to the concentration quenching effects. The critical distance R_c_ between the Dy^3+^ ions can be represented by Blasse [42],

(3) $$R_{c}=2{\left(3V/4\pi x_{c}Z\right)}^{1/3}$$

where *V* is the volume of the unit cell, *x_c_* is the critical concentration of Dy^3+^ ions, and *Z* is the number of host cations in the unit cell. For the BaWO_4_ host, V=399.032 Å, $x_{c}=0.07$, and Z=8. Therefore, *R_c_* was estimated to be about 11.08 Å. It is well known that there are three types of interactions involving electric multipole interactions in energy transfer: dipole–dipole, dipole–quadrupole, and quadrupole–quadrupole.

### 3.2. The Properties of RE^3+^ Doped BaWO_4_ Phosphors 

**Figure 5:**
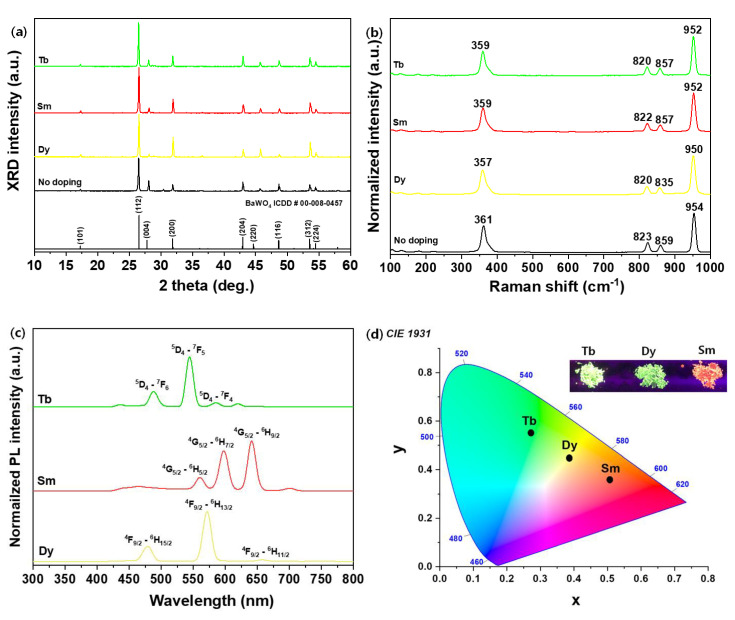
BaWO_4_ phosphors doped with different activator ions (Dy^3+^, Sm^3+^, Tb^3+^): (**a**) XRD patterns, (**b**) Raman spectra under a 633 nm laser, (**c**) photoluminescence spectra at 325 nm, (**d**) International Commission on Illumination (CIE) coordinates and inset phosphor pictures under a UV lamp.

The BaWO_4_ phosphors doped with Dy^3+^ showed structural and photoluminescent properties. In addition, synthesis of Tb^3+^-doped and Sm^3+^-doped BaWO_4_ phosphors used the same conditions as above (7 mol % rare-earth, 900 °C sintering temperature). Figure 5a shows XRD patterns and Figure 5b shows Raman spectra of the BaWO_4_ and rare-earth (RE: Dy^3+^, Sm^3+^, Tb^3+^)-doped BaWO_4_ phosphors. There were no phases detected for the activator ions, responding that the RE ions had invalidity on the BaWO_4_ phase composition but slightly changed the position where the main peak (112) plane was detected compared to the undoped BaWO_4_ powders. However, the lattice constant changed throughout the main peak (112) plane (no doping, d_(112)_ = 3.363 Å, Dy^3+^ d_(112)_ = 3.354 Å, Sm^3+^ d_(112)_ = 3.355 Å, Tb^3+^ d_(112)_ = 3.356 Å). The ionic radii for the cations of the added RE elements are r (Dy^3+^) = 1.03 Å, r (Sm^3+^) = 1.08 Å, r (Tb^3+^) = 1.18 Å, r (Ba^2+^) = 1.142 Å, r (W^6+^) = 0.74 Å [43]. As mentioned while explaining the XRD patterns in Figure 3a, these RE ions are expected to replace Ba^2+^ ions in the BaWO_4_ structure. To check the feasibility of this conjecture, Raman spectra of the powders were obtained as shown in Figure 5b. There are two types of vibration modes: internal, and external vibration, when considering the Raman active mode of the scheelite type xWO_4_ compound. The first correlate normal mode, with atoms inside the [WO_4_]^2−^ two tetrahedron, and the second is related to the oscillation of the WO_4_ tetrahedron around the divalent x atom. The classification of the tetragonal scheelite (BaWO_4_) primitive cells at wavevector k = 0, as theory calculation predicts 26 vibration, which can be expressed as (4) [44,45]:

(4) $$\Gamma =3A_{g}+5A_{u}+5B_{g}+3B_{u}+5E_{g}+5E_{u}$$

where all of the Raman-active modes vibrations (Ag, Bg, and Eg); A, B modes are non-degenerate, the E modes are twice as degenerate. The sub-fixed g and u represent even and odd, respectively, and represent the parity in the inverted state in a centrosymmetric crystal. The Au and Eu are acoustic modes matched to the zero frequency. The rest of these modes are the optical modes. The first member (g) in the materials of the scheelite structures belongs to the Raman activation mode. The second member (u) is only active at infrared (IR) frequencies. On the other hand, the B_u_ silent mode is not activated at that frequency. The 13 zone-center Raman-active modes predictable in the BaWO_4_ are described by the following equation [46].

(5) $$\Gamma =3A_{g}+5B_{g}+5E_{u}$$

According to the previous study, the Raman spectrum of the tungstates observed two types vibrational spectra as external and internal mode. Firstly, the lattice phonon called the rigid molecular units, which indicates the frequency of Ba^2+^ cations. Secondly, considering the fixed center of mass, it refers to the vibration inside the [WO_4_]^2−^ molecular units [46]. In this work, the E_2g_ mode positioned at two strong signals was monitored at 361 and 954 cm^−1^, and two weak signals were monitored at 823 and 859 cm^−1^ of the BaWO_4_ powders. As can be seen in figures, all Raman spectra of BaWO_4_ and RE-doped BaWO_4_ powders obtained in this work have tetragonal structure characteristics, which are consistent with the literature [46]. However, RE-doped BaWO_4_ powders shifted to low frequency. From the Raman spectrum mode, inversely proportional to the square root of the atomic mass, a shift to a low wavenumber in the Raman spectrum indicates substitution in cooperation with heavy RE atoms to the BaWO_4_ lattice, which is consistent with the conjecture resulting from the observations of the d_(112)_ lattice constant of XRD data. Figure 5c shows PL spectra of RE-doped BaWO_4_ powders under the UV wavelength (λ_ex_ = 325 nm). The PL spectra of the BaWO_4_:Dy^3+^, BaWO_4_:Sm^3+^, and BaWO_4_:Tb^3+^ powders synthesized with the same doping composition of the activator ions. The emission spectra for the first BaWO_4_:Dy^3+^ phosphors under ultraviolet excitation consisted of a strong yellow band centered at 572 (^4^F_9/2_ → ^6^H_13/2_) nm and two weak bands at 479 (^4^F_9/2_ → ^6^H_13/2_) and 658 (^4^F_9/2_ → ^6^H_13/2_) nm. The second BaWO_4_:Sm^3+^ phosphors’ red band was centered at 640 (^4^G_5/2_ → ^6^H_9/2_), 598 (^4^G_9/2_ → ^6^H_7/2_), and 560 (^4^G_9/2_ → ^6^H_5/2_) nm. Finally, the BaWO_4_:Tb^3+^ phosphors’ green band was centered at 544 (^4^D_4_ → ^6^F_5_) nm as well as two weak bands at 488 (^4^D_4_ → ^6^F_6_) and 640 (^4^D_4_ → ^6^F_4_) nm. The International Commission on Illumination (CIE) color coordinates, shown in Figure 5d, indicate that three distinct colors were possible for RE-doped BaWO_4_ phosphors.

**Figure 6:**
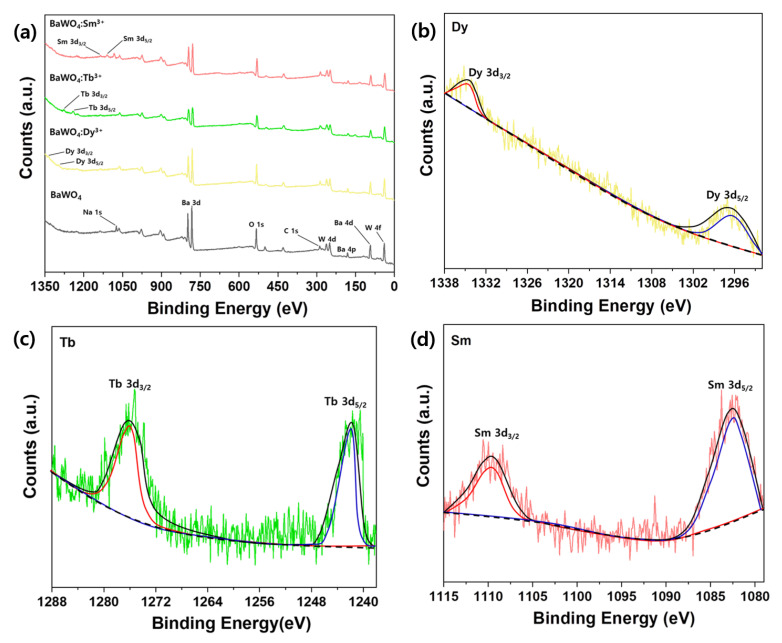
XPS spectra; (**a**) survey scan of un-doped BaWO_4_ powders and RE^3+^-doped BaWO_4_ phosphors, (**b**) scan of Dy^3+^ 3d states, (**c**) scan of Tb^3+^ 3d states, (**d**) scan of Sm^3+^ 3d states.

The chemical states of elements in the undoped BaWO_4_ and RE^3+^-doped BaWO_4_ powders were investigated by X-ray photoelectron spectroscopy (XPS) and are presented in Figure 6.

The survey scan of the undoped BaWO_4_ and RE^3+^-doped BaWO_4_ samples is shown in Figure 6a. The Ba 3d spectra consist of two peaks at 782 and 797 eV, corresponding to the 3d_5/2_ and 3d_3/2_ peaks (Ba), respectively. The W 4f spectra consisted of two peaks at 36 and 41 eV, which monitored the W 4f_7/2_ and W 4f_5/2_ peaks, respectively. The Ba/W atomic ratio was 1.02 and almost consistent with the initial stoichiometric molar ratio. The O 1s spectrum peak occurred at 533 eV. The oxidation atomic ratios (Ba/O, W/O) for the undoped BaWO_4_ powders were 0.25 and 0.24. These calculated ratios were steady with the literature value of 0.25% [47]. Using the O 1s component and C 1s at 288 eV in the BaWO_4_ sample showed a C/O atomic ratio of 0.58. The ratio value may be carbonate. The Na 1s peak was detected at 1074 eV. As a result of detection from the sodium tungstate reagent, it is thought that a small amount of sodium remained when washing was performed during precursor preparation. RE^3+^ 3d spectra were observed with RE^3+^-doped BaWO_4_ samples as shown in Figure 6b–d. Dy^3+^ 3d spectra were observed at about 1297 and 1340 eV, Tb^3+^ 3d spectra were observed at about 1241 and 1275 eV, and Sm^3+^ 3d spectra were observed at about 1082 and 1110 eV, which can be assigned to RE^3+^ 3d_5/2_ and 3d_3/2_ states based on Dy–O, Tb–O, and Sm–O bonding [48]. The RE ions can be evaluated oxidation state as +3. The appearance of RE^3+^ ions in the BaWO_4_ lattice is significant for luminescence.

### 3.3. RE^3+^ Doped BaWO_4_ Phosphors for Anti-Conterfeiting Applications

**Figure 7:**
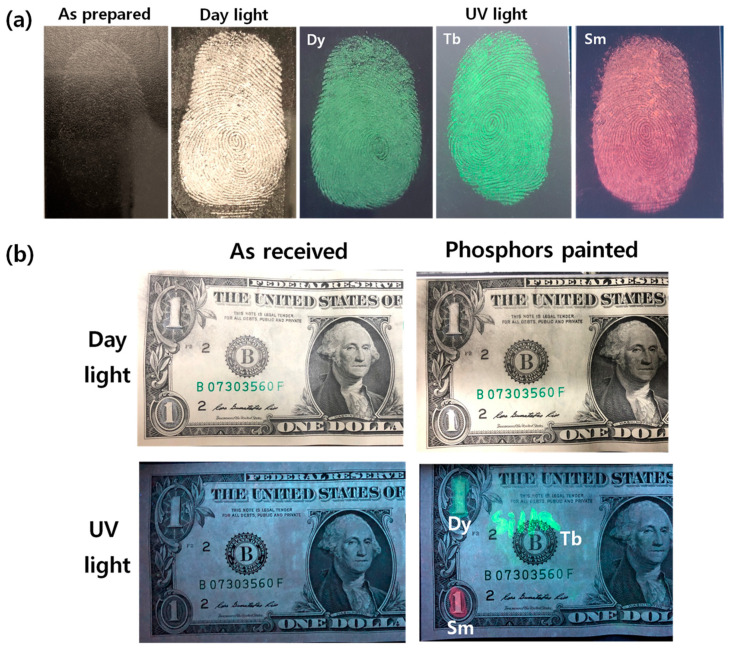
Images of phosphors (**a**) fingerprinted on glass substrates and (**b**) painted on the US dollar bank notes in daylight and under UV light.

The fingerprints of three people developed by RE^3+^-doped BaWO_4_ phosphors on the glass surface are shown in Figure 7a. The donor images, bare images, and powdered images used RE^3+^-doped BaWO_4_ phosphors, and fluorescent images were taken under a UV lamp. The shape of the obtained fingerprints from one person has a whorl loop. The naked image is blurred in daylight. Because the particles adhere to the moisture component in the fingerprint, the powder image using the prepared phosphor is very eye-catching. Under ultraviolet light, the fluorescent image shows red and green emission colors, which can confirm that the contrast and resolution of the fingerprint have been improved. The BaWO_4_ powders with doped RE were dispersed in an aqueous solution of PVP to prepare a nebulous colloidal solution. The colloidal solution of RE-doped BaWO_4_ phosphors painted on the surface of a bank note are shown in Figure 7b. 

In daylight, it is hard to distinguish between the bare bank note and phosphors-coated bank note with the naked eye due to the transparency of the phosphors’ thin film and text. However, intense yellow and red colors from phosphors film and green text “Silla” can be seen under the irradiation of UV light. As a result, the RE-doped BaWO_4_ phosphor emits optical transparency and visible light under UV radiation, so that it can be hidden and visually recognized in a normal environment, which is an essential element for anti-counterfeiting ink applications. RE-doped BaWO_4_ particles can be considered as promising candidates for luminescent labels suitable for anti-counterfeiting applications due to their good transparency and luminescence characteristics.

## 4. Conclusions

BaWO_4_ particles and BaWO_4_:RE^3+^ (RE = Dy, Tb, Sm) phosphors were described with various sintering temperatures and quantities of rare-earth ions via co-precipitation. The BaWO_4_ characteristics of the samples were inspected through XRD, XPS, FE-SEM, Raman spectroscopy, and photoluminescence instruments. The XRD patterns of all samples as shown, in spite of the kind and concentration of the rare-earth ions, included phases of BaWO_4_. For the RE^3+^-doped BaWO_4_ phosphors, the crystalline monitored a tendency to agglomerate into shingle-like shapes. The dominant emission spectra of rare-earth ion-doped BaWO_4_ phosphors showed yellow (Dy^3+^), green (Tb^3+^), and red (Sm^3+^) emissions. The solution-based coating of RE-doped BaWO_4_ phosphors on bank notes showed the good transparency of the thin film, with color emission. As a result, rare-earth doped BaWO_4_ phosphors tunable colors as yellow, green, and red emission materials for the development of anti-counterfeiting.
